# Neuro-schistosomiasis with palm tree contrast enhancement pattern, a report of three cases, and review of literature

**DOI:** 10.1259/bjrcr.20200053

**Published:** 2020-10-08

**Authors:** Ahmed El Beltagi, Khalid Salem, Mohamed Hanoun

**Affiliations:** 1 Division Neuroradiology, Neuroscience Institute, Hamad Medical Corporation, Weill Cornell Medicine, Doha, Qatar

## Abstract

We describe three cases of neuroschistosomiasis, two cases with cerebral schistosomiasis due to *Schistosoma japonicum*, with multiple pseudotumoral lesions presented with seizures and hemiparesis respectively, and a spinal cord conus medullaris schistosomiasis due to *Schistosoma mansoni* presented with conus medullaris syndrome.

In the two cases with cerebral schistosomiasis imaging with CT revealed multiple areas of brain edema, and gyriform calcifications in both cerebral hemispheres, which suggested cerebral parasitemia, chronic venous hypertension, multifocal cerebral vascular malformation, or a forme fruste Sturge Weber syndrome. Further MRI revealed corresponding blooming, T2W (weighted) -FLAIR (fluid attenuated inversion recovery) ibright signal intensity and enhancing lesions. In the third case with spinal cord involvement MRI revealed signal abnormality on T1W and T2W images with patchy and punctate post i.v. contrast enhancement of the conus medullaris.

Excision biopsy and histopathological examination were undertaken for the first brain case and spinal cord case and showed multiple schistosomal granulomas in different evolution phases. In the second brain case, the diagnosis was suggested based on our experience with prior cases, positive laboratory tests, and urinary bladder wall biopsy.

Neuroschistosomiasis must be considered in the differential diagnosis of multiple cerebral calcifications, and multiple nodular and linear like lesions with characteristic arborized enhancement pattern, especially for patients coming from endemic areas for Schistosomiasis.

## Introduction

Schistosomiasis is a parasitic infection that uses aquatic clams as intermediate hosts, the male and other mammals as definitive hosts. Five species of *Schistosoma* are capable of causing disease in humans: *S. mansoni, S. japonicum, S. haematobium, S. intercalatum* and *S. mekongi*.^1^ Schistosomiasis affects about 200 million individuals worldwide and are endemic in Brazil, Suriname, Venezuela, several of the Caribbean Islands, equatorial west and center-south of Africa, and Egypt.^2^ Yet, because of current easiness of locomotion and migration, schistosomal infection became more frequently found in non-endemic countries.^3,4^

Neuroschistosomiasis with involvement of the central nervous system (CNS) include the brain and spinal cord, with spinal cord being most frequentlyaffected by *S. mansoni*, and cerebral schistosomiasis (CS) being most frequently affected by *S. japonicum*. Two forms of neuroschistosomiasis have been described, the tumoral (pseudotumoral) and diffuse pseudotumoral forms. The corresponding imaging findings of the pseudotumoral form include a single or multilobar cerebral involvement, with rare cerebellar, basal ganglia, and mid-brain involvement, the diameter of these lesions range from <1 cm to about 7 cm, with CT parenchymal calcification, and MRI long T1 and T2 signals, prominent post-contrast enahancement, and surrounding vasogenic edema and mass effect. The diffuse pseudotumoral form comprises multiple discrete 3 mm intensely enhancing nodules clustered together in a large mass.^1,5–7^

## Case 1

**Figure 1.**
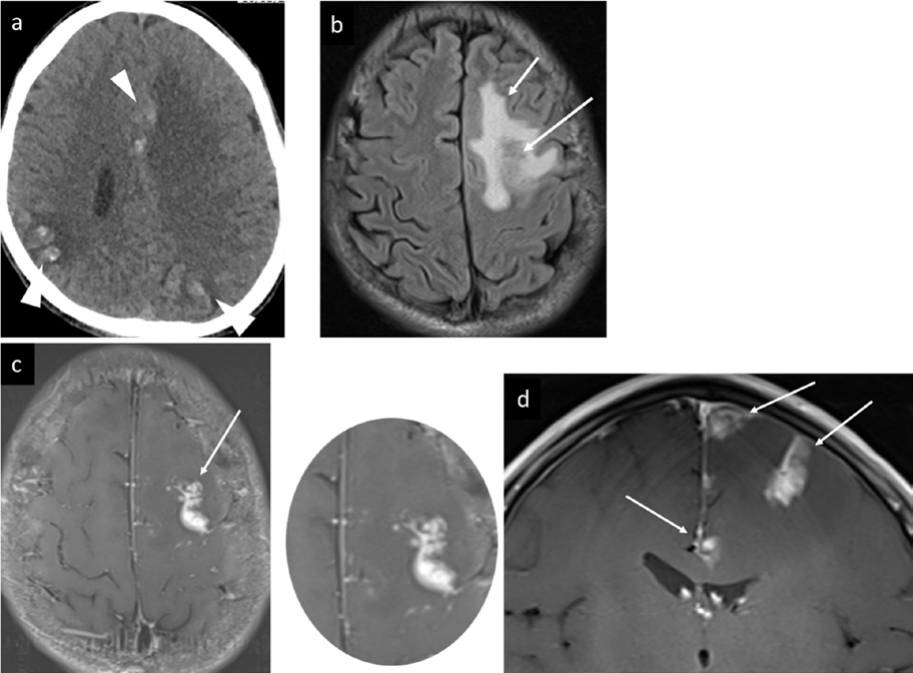
Case 1 brain schistosomiasis, (a) axial non-contrast CT at ventricular level showing multiple cortical and subcortical gyriform linear, and nodular hyperdensities in parietal and left frontal lobes bilaterally (arrowheads), with significant perilesional vasogenic oedema, (b) axial FLAIR-*T*_2_Wimage showing gyriform low signal (long arrow) with perilesional hyperintense vasogenic oedema (short arrow) in the left frontal lobe lesion, (c) axial with zoomed-in and (d) coronal *T*_1_W post i.v. contrast image showing branching linear and nodular enhancements forming arborized appearance, simulating palm tree leaves (arrows). FLAIR, fluid attenuated inversion recovery.

A 25-year-old male Filipino patient came to the A&E department complaining of seizures, his laboratory blood tests were normal. Non-contrast CT scan of the head showed multiple bilateral cerebral cortical and subcortical hyperdense linear and nodular lesions, consistent with parenchymal calcifications, surrounded by hypodense vasogenic edema, involving the left frontal, left occipital and bilateral parietal lobes (Figure 1a).

MRI was then undertaken for better characterization of the parenchymal abnormalities and showed multiple lesions with linear and nodular post i.v. contrast enhancement surrounded by areas of vasogenic edema (Figure 1b, c and d).

Excision brain biopsy was done, and pathological examination of the specimen showed granuloma formation around characteristic *Schistosoma japonicum* ova with absence of protruding spine.

## Case 2

A 25-year-old Filipino lady, presented to the emergency department complaining of a 1 day history of left-sided lower limb weakness causing walking difficulty, associated with moderate headache radiating to the back of the neck, but otherwise no other associated symptoms.

She was admitted to the hospital, where she had tonic clonic seizures followed by confusion, headache, and vomiting. Her past medical history was unremarkable.

**Figure 2.**
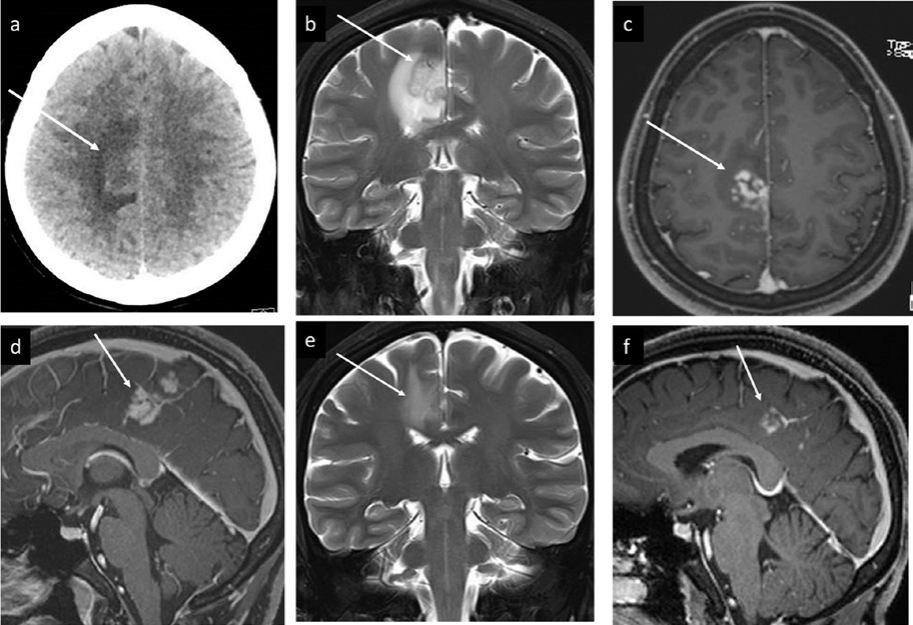
Case 2 Brain schistosomiasis, (a) Plain CT head showing left frontal parasagittal cortical swelling, and subcortical vasogenic oedema (arrow), (b) MRI Coronal *T*_2_W image,showing corresponding Gyral swelling low signal, and surrounding subcorticalvasogenic edema, (c, d) sagittal, and axial post-contrast T1W image respectively,showing the typical arborized palm tree-like enhancement. 2 weeks Post-treatment follow-up MRI showed dramatic improvement with decreased edema on coronal *T*_2_W image (e), and decreased enhancement on sagittal post-contrast *T*_1_W image (f).

Plain CT head showed left frontal parasagittal cortical swelling with mild increased density and associated subcortical vasogenic edema (Figure 2a). MRI scan of the brain showed a frontal cortical lesion with typical arborized branching linear and nodular post contrast enhancemment pattern, surrounded by vasogenic edema (Figure 2b, c and d), which was highly suggestive of schistosomiasis, with differential consideration of brain primary or metastasis being less likely.

Plain CT scan of the chest, abdomen, and pelvis (not shown) was also undertaken as a workup of possible malignancy with brain metastasis. And showed nodular urinary bladder wall and calcified capsular liver lesions suggestive of chronic schistosomiasis. The patient denied any urinary or gastrointestinal symptoms, but she gave history that she used to play and drink from local river back home in her childhood.

Schistosoma antibody (Ab) titer was positive at 1:160. And urinary bladder wall biopsy was consistent with schistosomiasis. Given the above imaging and clinical background the diagnosis of neuroschistosomiasis of the brain was entertained, and treatment for schistosomiasis was started, and she received a single dose 40 mg/kg Praziquantel, and she was discharged on oral levetiracetam (Keppra) and prednisolone for seizure control.

On 2 weeks clinical follow-up visit, the patient showed dramatic clinical improvement, and follow-up MRI showed significant resolution of the enhancing brain lesions, and decreased vasogenic edema (Figure 2e and f).

## Case 3

**Figure 3.**
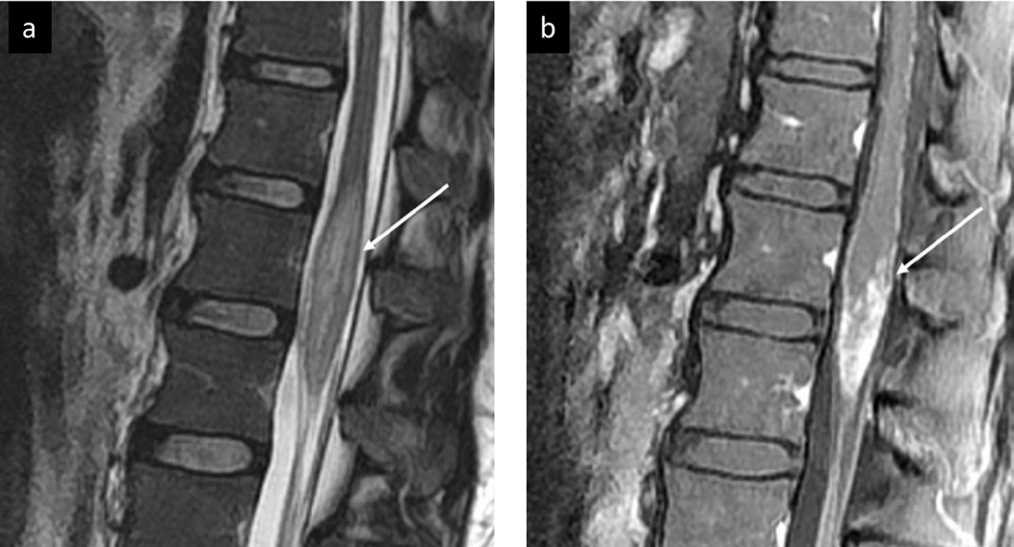
Case spinal cord schistosomiasis, (a) sagittal *T*_2_W image of the lower dorsal and upper lumbar spine showing the expansion of the conus medullaris with a patchy area of high signal intensity (arrow), (b) sagittal *T*_2_W post i.v. contrast image shows nodular and linear enhancement forming arborized appearance (arrow), note the increased enhancement of the intrathecal nerve roots.

A 29-year-old male Egyptian patient presented with acute lower limb numbness and weakness with incontinence of urine and stool. MRI of the dorsolumbar spine showed moderate swelling of the lower spinal cord and conus medullaris, abnormal bright signal intensity on *T*_2_W image MRI, and thick nodular and linear enhancement on post i.v. contrast *T*1W image MRI (Figure 3). The patient underwent microlaminectomy with surgical biopsy, and histopathological examination of the specimen showed multiple granulomas around *S. mansoni* ova with typical lateral spines.

## Discussion

Neuroschistomiasis (NS) confined to cerebral areas, has no definite lobe predilection. With more clinically significant NS include involvement of the spinal cord and cerebellum..^7,8^

The clinical syndrome of spinal NS is usually an acute or subacute myelopathy, with or without polyradiculitis, causing lumbosacral and lower limbs pain, paraparesis or paraplegia, bowel, and bladder sphincter dysfunction, and sensory, sexual and reflexic disturbances.^9^

Whereas brain involvement may lead to headaches, nausea and vomiting, papilledema, seizures, or motor deficits according to the location of the granuloma.^9^

CT in patients with brain schistosomiasis, show single or multiple variably enhancing hyperattenuated lesions with surrounding hypoattenuated oedema may be present.^10^

Contrast-enhanced *T*_1_ W MRI reveals a central linear enhancement surrounded by multiple enhancing punctate and nodular lesions, forming a characteristic “arborized” appearance. Pathologically, this enhancement pattern has been correlated with a host granulomatous response to Schistosomal ova. This pattern of enhancement though characteristic of brain schistosomiasis, is not present in all cases, but when observed the diagnosis of CNS schistosomiasis should be strongly considered.^11^

Additional findings related to liver dysfunction and portosystemic shunt may be seen as bilateral symmetric *T*_1_W hyperintense signal of the globus pallidus and also, in substantia nigra in case of hepatic schistosomiasis japonicum even in the absence of liver dysfunction believed to be related to manganese deposition. NS findings in children and young adults with hepatosplenic schistosomiasis also include T2W cerebral white matter hyperintensity with rare corpus callosum involvementat.^10^

In our two brain cases, CT showed multifocal and multilobar involvement, which was bilateral in the first case, and unilateral in the second case, cerebral cortical and subcortical hyperdense linear and nodular lesions, consistent with parenchymal calcifications, surrounded by hypodense vasogenic edema. MRI was then undertaken for better characterization of the parenchymal abnormalities and showed multiple lesions with linear and nodular enhancement surrounded by areas of vasogenic edema (Figures 1 and 2).

Thaigo et al,^6^ described multiple brain lesions with a unique multinodular appearance in two cases of cerebral schistomiasis, which were almost similar to our first two cases. In the first case in this study, cranial CT showed multiple hyperdense lesions with surrounding hypodensity, and post-contrast enhancement in the left temporal lobe, thalamus, and pons, in the next case cranial CT showed a hypodense lesion surrounding a right parieto-occipital area of diffuse parenchymal calcifications, brain MRI showed the multinodular aspect of these lesions with surrounding vasogenic edema and mass effect and revealed an additional lesion in the left cerebellum in the next case.

*Júnior* et al in another study^9^ described a similar multinodular tumefactive lesion in the left temporal lobe, which showed remarkable regression on follow-up MRI 3 months after treatment with praziquantel.

**Figure 4.**
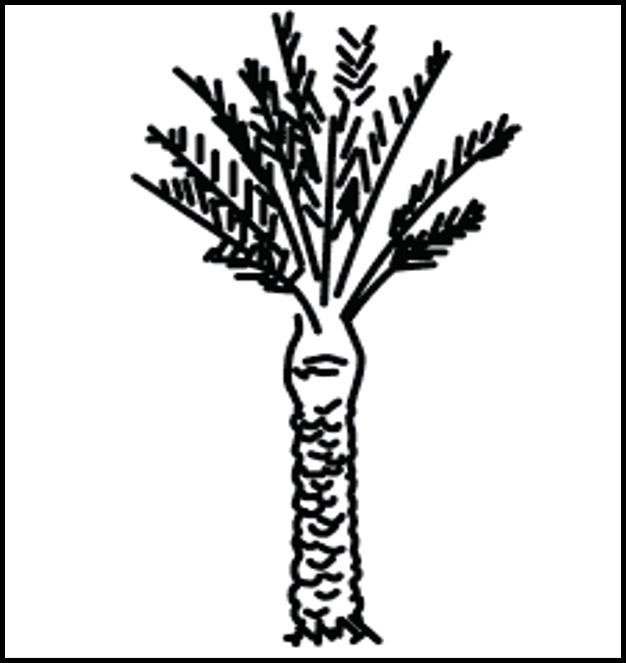
Drawing of a date palm tree. The gyral enhancement is likened to the palm tree stem andthe arborized linear and nodular pattern is likened to the leaves of the palmtree.

*Sanelli et al*^11^ described two cases of cerebral schistosomiasis involving the right anterior parietal lobe in one and the left temporal lobe in another, both appeared as mass-like lesions**,** where the temporal lobe lesion showed typical (arborized) linear and nodular enhancement similar to our first two cases, which we suggest can be likened to palm tree and leaves (Figure 4).

MRI of spinal cord schistosomiasis often shows mild to moderate expansion with the predilection to the lower spinal cord and conus medullaris.

Three different forms of abnormal intraspinal contrast enhancement have been described^1^: intramedullary nodular enhancement,^2^ Peripheral enhancing lesions on the cord surface.^3^ Enhancing thickened nerve roots and cauda equina^12^.

*Sanelli et al*,^11^ described a case of spinal cord schistosomiasis with findings similar to our case; with expansion of the distal spinal cord and conus medullaris, diffuse T2 signal prolongation, central linear enhancement surrounded by multiplepunctate nodules which gave a clustered appearance enhancement pattern on T1 post i.v. contrast series.

*Salim et al*^13^ described spinal cord involvement in five patients, with the following patterns: multiple intramedullary nodular enhancement which varied in size from minute foci of <3 mm in diameter to larger nodules up to 20 mm, peripheral enhancing lesions located mainly on the anterior aspect of the cord, a feature resembling our third index case, solitary enhancing mass-like lesion, and thickened enhancing nerve roots and cauda equina, in addition, a small reactional syrinx with non-enhancing walls was detected proximal to the lesion in one patient.

## Conclusion

Our three cases with cerebral, and spinal cord schistosomiasis, demonstrated similar characteristic imaging appearances with other studies in literature, with characteristic arborized linear and nodular enhancement pattern, which we suggest can be likened to palm tree and leaves. And, we believe like others that in the proper clinical settings such appearances should obviate the need for biopsy and should indicate induction of treatment with early response assessment by imaging at 2 weeks showing a decrease in edema and post-contrast enhancement and follow-up imaging by 3 months showing significant recovery of brain lesions..

## Learning points

- Schistosomiasis is one of the parasitic infections which can affect the CNS (Neuro-Parasitemia).
- Neuroschistosomiasis has a characteristic appearance on CT and MRI which should in the proper clinical context lead to further laboratory confirmation and obviate the need for an invasive surgical biopsy.
- Early 2 weeks post anti-schistosomal treatment imaging assessment and subsequent imaging follow up at 3 months can further confirm the diagnosis by showing adequate treatment response.
